# Inhibition of interferon I induction by non-structural protein NSs of Puumala virus and other vole-associated orthohantaviruses: phenotypic plasticity of the protein and potential functional domains

**DOI:** 10.1007/s00705-021-05159-y

**Published:** 2021-08-13

**Authors:** Florian Binder, Giulia Gallo, Elias Bendl, Isabella Eckerle, Myriam Ermonval, Christine Luttermann, Rainer G. Ulrich

**Affiliations:** 1 Friedrich-Loeffler-Institut, Federal Research Institute for Animal Health, Institute of Novel and Emerging Infectious Diseases, Greifswald-Insel Riems, Germany; 2 Department of Virology, Institut Pasteur, Antiviral Strategies, Paris, France; 3 University of Bonn, Medical Centre, Bonn, Germany; 4 Friedrich-Loeffler-Institut, Federal Research Institute for Animal Health, Institute of Immunology, Greifswald-Insel Riems, Germany; 5 Present Address: University Hospital Freiburg, Institute of Virology, Freiburg, Germany; 6 Present Address: Geneva Centre for Emerging Viral Diseases, Division of Infectious Diseases, University Hospital of Geneva, Geneva, Switzerland

## Abstract

The orthohantavirus Puumala virus (PUUV), which is transmitted by bank voles (*Clethrionomys glareolus*), and other vole-borne hantaviruses contain in their small (S) genome segment two overlapping open reading frames, coding for the nucleocapsid protein and the non-structural protein NSs, a putative type I interferon (IFN-I) antagonist. To investigate the role of NSs of PUUV and other orthohantaviruses, the expression pattern of recombinant NSs constructs and their ability to inhibit human IFN-I promoter activity were investigated. The NSs proteins of PUUV and related cricetid-borne orthohantaviruses showed strong inhibition of IFN-I promoter induction. We identified protein products originating from three and two methionine initiation codons in the NSs ORF of PUUV during transfection and infection, respectively. The three putative start codons are conserved in all PUUV strains analysed. Translation initiation at these start codons influenced the inhibitory activity of the NSs products, with the wild-type (wt) construct expressing two proteins starting at the first and second methionine and showing strong inhibition activity. Analysis of *in vitro*-generated variants and naturally occurring PUUV NSs proteins indicated that amino acid variation in the NSs protein is well tolerated, suggesting its phenotypic plasticity. The N-terminal 20-amino-acid region of the NSs protein was found to be associated with strong inhibition and to be highly vulnerable to amino acid exchanges and tag fusions. Infection studies using human, bank vole, and Vero E6 cells did not show obvious differences in the replication capacity of PUUV Sotkamo wt and a strain with a truncated NSs protein (NSs21Stop), showing that the lack of a full-length NSs might be compensated by its N-terminal peptide, as seen in transfection experiments. These results contribute to our understanding of virus-host interactions and highlight the importance of future innate immunity studies in reservoir hosts.

**Supplementary Information:**

The online version contains supplementary material available at 10.1007/s00705-021-05159-y.

## Introduction

**Fig. 1:**
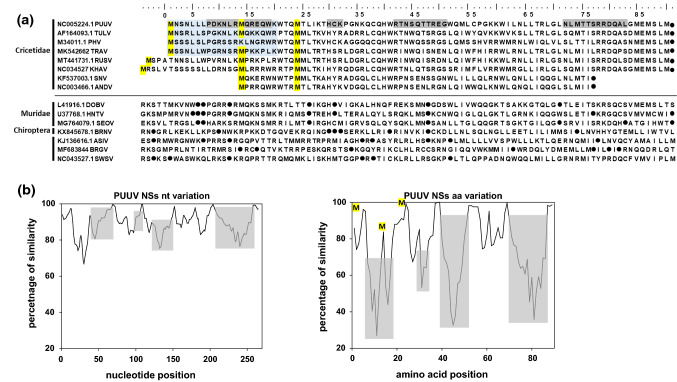
Sequence comparison of putative hantavirus NSs proteins and the coding regions (**a**), and SimPlot analysis of NSs nucleotide and amino acid sequences of 78 PUUV strains from all clades compared to the PUUV Sotkamo wt strain (**b**). **a** Sequence alignment of the putative NSs proteins of cricetid-associated hantaviruses and the corresponding region of other hantaviruses of Muridae, Chiroptera, and Eulipotyphla hosts. Conserved methionine (start) codons are shown in yellow, the most divergent protein regions are highlighted in grey, and stop codons are indicated by a dot (●). The region of NSs shown to be important for IFN-I promoter inhibition in our analyses is highlighted in blue. The accession numbers are indicated on the left of the NSs sequences of the different orthohantaviruses used for the analysis, which are as follows: PUUV, Puumala virus (strain Sotkamo); TULV, Tula virus (strain g20-s); PHV, Prospect Hill virus; TRAV, Traemmersee virus; RUSV, Rusne virus (strain LT15/299); KHAV, Khabarovsk virus (strain Fuyuan); SNV, Sin Nombre virus (strain 77734); ANDV, Andes virus (strain Chile-9717869); DOBV, Dobrava-Belgrade virus (genotype Dobrava 3970/87); HTNV, Hantaan virus (strain CUMC-B11); SEOV, Seoul virus (strain SEOV/NL/Rn2147/2016); BRNV, Brno virus (strain 7/2012/CZE); ASIV, Asikkala virus (strain Asikkala); BRGV, Bruges virus (strain DE/Wandlitz/TE/2013/1); SWSV, Seewis virus (strain EWS25). Of note, in non-cricetid-associated hantaviruses, the region shown did not contain a start codon and contained multiple stop codons within the region corresponding to the NSs ORF in cricetid-borne hantaviruses. **b** SimPlot analysis of NSs nucleotide and amino acid sequences of 78 PUUV strains from all clades, obtained from GenBank with PUUV Sotkamo wt strain (NC_005224.1) as a query. For SimPlot analysis with a window size of 9 nucleotides (nt)/3 amino acids (aa) and a step size of 3 nt/1 aa, scripts were written in R [26]. The three putative start (methionine) codons of PUUV NSs are depicted in yellow at the corresponding peak of the highest sequence conservation, and the most divergent amino acid regions are shown in grey. An additional multiple alignment of NSs amino acid sequences of PUUV strains of all known clades is shown in Supplementary Figure S3

Rodent-borne orthohantaviruses belong to the family *Hantaviridae* in the order *Bunyavirales* and are distributed all over the world. Infections with pathogenic orthohantaviruses can cause hemorrhagic fever with renal syndrome (HFRS) or hantavirus cardiopulmonary syndrome (HCPS) in humans [1]. In Europe, Puumala virus (PUUV) is responsible for most HFRS cases. Bank voles (*Clethrionomys glareolus*, syn. *Myodes glareolus*), which are widely distributed in Europe and parts of Asia, act as reservoir hosts of this virus [2]. The small (S) segment of most bunyaviruses, in addition to the nucleocapsid (N) protein, encodes a non-structural (NSs) protein in an overlapping or antisense open reading frame (ORF) [3]. The ORF encoding NSs is only present in orthohantaviruses associated with rodents of the family Cricetidae (voles, lemmings, and New World rats/mice) and not in those associated with other hosts (Fig. 1a; [4]). The orthohantavirus NSs protein is expressed from a +1 overlapping ORF in the N protein mRNA via a leaky scanning mechanism, initiated at the first AUG codon after the N protein start codon [5]. The NSs proteins of PUUV and the orthohantavirus Tula virus (TULV) have been suggested to be functional and act as weak type I interferon (IFN-I) inhibitors in immortalized monkey CV-1 (COS-7) cells [6]. For TULV and the orthohantavirus Andes virus (ANDV), NSs proteins have been reported to accumulate early in infection in the cytoplasm and in the perinuclear area, where they interact with factors of the innate immune system [5, 7]. A recent study confirmed the inhibitory activity of the NSs proteins of PUUV, TULV, and the orthohantavirus Prospect Hill virus (PHV) but also suggested the involvement of other viral proteins in countermeasures to the cellular antiviral response [8]. Arthropod-borne bunyaviruses such as the phlebovirus Rift Valley fever virus (RVFV) and the orthobunyavirus Bunyamwera virus (BUNV) express well-studied NSs proteins as their main virulence factors, inhibiting transcription and protein synthesis in host cells [9, 10]. In the case of BUNV, the orthobunyavirus La Crosse virus (LACV), RVFV, and the phlebovirus Toscana virus (TOSV), the NSs proteins inhibit the IFN-I system by blocking RNA polymerase II transcription or by degradation of dsRNA-dependent protein kinase (PKR) [9, 11–15].

Interactions of the host immune system with invading viruses are of outstanding importance for susceptibility, transmission, and outcome of viral infections in host organisms. Viruses invading a host are detected at the host-pathogen interface by the innate immune system early in infection [16, 17]. Pattern recognition receptors (PRRs) interact with conserved structural motifs, called pathogen-associated molecular patterns (PAMPs), displayed by infectious agents. PRRs activate factors of the innate immune response such as IFN-I and pro-inflammatory cytokines, which impair virus replication and induce long-term immune responses. IFN-I expression is tightly controlled by latent transcription factors, which are activated upon recognition of intruding viruses by cytoplasmic PRRs that sense viral double-stranded RNA, such as retinoic acid inducible gene I (RIG-I) or melanoma differentiation antigen 5 (MDA-5). The activating part of RIG-I, the caspase recruitment domain (CARD), initiates signaling through tumor necrosis factor (TNF) receptor associated factor 3 (TRAF3), TRAF-family member-associated NFκB activator-binding kinase 1 (TBK-1), and IκB-kinase ε (IKKε), leading to phosphorylation of transcription factors such as interferon regulatory factor 3 (IRF-3) and subsequent IFN-β synthesis [16]. The synthesized IFN-I induces a crucial host defense mechanism by activating immune cells or effector proteins such as myxovirus resistance (Mx) protein [18].

Here, we investigated the expression pattern of wild-type and modified hantaviral NSs proteins after transfection and infection. We also investigated the RIG-I signaling cascade for activation of the IFN-β promoter by different wild-type orthohantavirus-derived NSs proteins and PUUV NSs proteins from wild bank voles of different geographic origin, as well as the influence of introduced mutations on the expression pattern and activity of PUUV NSs variants. Finally, we investigated the role of NSs in viral growth in human and bank vole cells, using cell-culture-derived wild-type (wt) PUUV and a mutant strain with a stop codon at position 21 (NSs21Stop). This mutant virus emerged spontaneously in Vero E6 cells infected with PUUV Sotkamo and was isolated by plaque purification. Sequence analysis revealed the presence of a stop codon mutation at position 21, but no further mutations were found in the coding sequence of the S segment compared to PUUV Sotkamo wt. The replication capacity of this mutant strain in bank vole reservoir cells had not been investigated before [19].

## Materials and methods

### Multiple sequence alignment and SimPlot analysis

NSs sequences of 78 PUUV strains from all PUUV clades obtained from GenBank NCBI (accession numbers: AB010730, AB010731, AB297665, AB433843, AB433845, AB675453, AB675463, AF063892, AF294652, AF367071, AF442613, AJ223369, AJ223371, AJ223374, AJ223375, AJ223380, AJ238790, AJ238791, AJ277030, AJ277031, AJ277033, AJ314598, AJ314599, AJ888751, AM695638, AY526219, AY954722, DQ016432, EU439968, FN377821, GQ339474, GQ339476, GQ339477, GQ339478, GQ339479, GQ339480, GQ339481, GQ339482, GQ339483, GQ339484, GQ339485, GQ339486, GQ339487, GU808824, GU808825, JN657228, JN657229, JN657230, JN657231, JN696358, JN696372, JN696373, JN696374, JN696375, JN831943, JQ319162, JQ319163, JQ319168, JQ319170, JQ319171, KJ994776, KT247592, KT247593, KT247595, KT247596, KT247597, L08804, M32750, U14137, U22423, Z21497, Z30702, Z30704, Z30705, Z46942, Z48586, Z69991, Z84204) were compared at the nucleotide and amino acid level with the NSs sequence of the PUUV Sotkamo wt strain (NC_005224.1) using BioEdit version 7.0.5.3.. The PUUV clades are defined according to Castel et al. [20]. For SimPlot analysis with a window size of 9 and a step size of 3 for nucleotide sequence analysis or a window size of 3 and step size of 1 for amino acid sequence analysis, scripts were written in R [21].

### Viruses and cells

Vero E6 and bank vole renal epithelial cells (MyglaSWRec.B, Western evolutionary lineage [22]) were grown in Dulbecco's modified Eagle medium (DMEM) containing 10% fetal calf serum (FCS). Human A549 cells were cultivated in Ham´s F12 medium with 10% FCS in 5% CO_2_ at 37 °C. Baby hamster kidney (BHK-21) and human embryonal kidney (HEK 293-T) cells were grown in modified Eagle´s medium supplemented with 10% FCS. PUUV wt strain Sotkamo or a cell-culture-derived PUUV Sotkamo variant (NSs21Stop) with a stop codon mutation at position W21 in the NSs ORF [19] were used for infection studies at a multiplicity of infection (MOI) of 0.5.

### Generation of polyclonal anti-NSs serum

For generation of a rabbit polyclonal antiserum, the NSs-ORF nucleotide sequence of wt PUUV strain Sotkamo was genetically fused between a 5′-6x his tag coding sequence and the coding sequence for bacterial lumazine synthase (LS) at the 3′ end, a protein previously described to be an efficient carrier for generation of high-titer antisera [23]. The 6xHis-NSs-LS complex was expressed in *Escherichia coli* and purified via Ni-NTA agarose (Thermo Fisher). Two rabbits were immunized and given a booster twice every 4 weeks until sera were collected 3 months after the initial immunization.

### Plasmids

RNA was extracted from cultured cells infected with PUUV strain Sotkamo wt or lung tissue of PUUV-infected bank voles from Baden-Wuerttemberg (BW) in southwest Germany and from North Rhine-Westphalia (NW) and the Osnabrück (OS) region of Lower Saxony, both in northwest Germany, using QIAzol Lysis Reagent (QIAGEN). Afterwards, the NSs-ORF of wt PUUV Sotkamo (HE801633.1) and the field strains from BW, NW, and the OS region (for details, see Supplementary Tables S1 and S2) were amplified via conventional RT-PCR using a Superscript III RT-PCR Kit (Thermo Fisher) with the primers 40f (5′-CTGGAATGAGTGACTTAAC-3′) and 393r (5′-CTCCAATTGTATACCAATCT-3′) and then cloned into the pCR2.1-TOPO plasmid using a TOPO TA cloning kit (Thermo Fisher). Restriction sites (underlined) were added to the NSs-ORF by PCR using the primers NSs BamHI fwd (5′ CAGGAGGATATAAGGATCCATGAACAGCAACTTA 3′) and NSs EcoRI rev (5′ CCTCTATGTCAATGGGAATTCCATCAAGG 3′). After restriction digestion of the RT-PCR products, the NSs-ORFs were inserted into pcDNA3 (Invitrogen) containing the coding sequence for a C-terminal hemagglutinin (HA)-tag at the 3′ end. NSs-ORFs of TULV (AF164093.1), ANDV (NC_003466.1), the orthohantavirus Sin Nombre virus (SNV, KF537003.1), PHV (M34011.1), and the orthohantavirus Khabarovsk virus (KHAV, NC_034527.1) were obtained as synthetic genes (Eurofins Genomics) and inserted into pcDNA3-HA in the same way as described above.

For reporter assays, the following plasmids were used: p125-FFluc, containing the human IFN-β promoter and firefly luciferase coding sequence; pcDNA3-RIG I (constitutively active N-terminal part) for activation of the human IFN-β signaling pathway; pRluc, encoding the *Renilla* luciferase under the control of a human cytomegalovirus (HCMV) immediate early (IE) promoter for monitoring transfection efficiency; pcDNA3 as a vector control, and the positive control plasmid pCR3-P, encoding rabies virus (RABV) phosphoprotein (P protein) [24].

### Mutagenesis and generation of NSs fusion proteins

Plasmids encoding the PUUV wt NSs protein were further modified using QuikChange Site-Directed Mutagenesis using Phusion High-Fidelity DNA Polymerase (Thermo Fisher). Stop codons were introduced at codon positions 2, 14, and 21 in the NSs ORF. To evaluate the functionality of alternative putative translation initiation codons, the methionine codons 1, 14, and 24 were substituted by alanine codons, both alone or in combination (1+14, 14+24, 1+14+24, Fig. 1a). Residues or regions of interest in the PUUV OS strain KS19/16 NSs plasmid were substituted by alanine (A) codons for evaluation of the plasticity of the NSs protein at codon positions 2-4 (NNN2-4(A)_3_), 11 (S11A), 16-19 (RRQW16-19(A)_4_), 21-23 (WTQ21-23(A)_3_), 25-28 (TLTR25-28(A)_4_), 31 (C31A), 38 (C38A), 49 (S49A), and 56 (C56A). Primer sequences are available upon request.

To investigate the expression of and potential influence of a C-terminal tag on the PUUV NSs 1-20 construct, either an HA tag or a foot-and-mouth disease virus (FMDV) 1A coding sequence were genetically fused C-terminally to PUUV Sotkamo NSs amino acids 1-20. The NSs 1-20-HA construct was generated by modification of the wt NSs-HA plasmid by inserting an EcoRI site after codon 20 and before the HA-tag coding sequence and subsequent EcoRI cleavage and religation. The FMDV 1A coding sequence was cloned from an existing 1A expression plasmid (Laboratory C. Luttermann) via the EcoRI and XbaI sites into construct NSs1-20-HA, thus replacing the HA-tag with the FMDV 1A coding region.

### Luciferase assays

For transfection of HEK 293-T cells in 6-well plates, a plasmid DNA mix of 0.5 µg of p125-FFluc, 0.005 µg of pRluc, 0.5 µg of pcDNA3-huRIG-I, and 1 µg of pcDNA3-HA plasmid containing one of the NSs coding sequences was made in 300 µl of Opti-MEM. The resulting plasmid DNA mix and 4 µl of Lipofectamine 2000 transfection reagent (Thermo Fisher) in 300 µl of Opti-MEM were mixed, incubated for 20 min at room temperature, and applied to the cells. After 3 h, the transfection mix was replaced by 1 ml of MEM + 10% FCS. Eighteen hours later, cell extracts were prepared, and luciferase activity was measured using the Dual-Luciferase Reporter Assay System (Promega) according to the manufacturer’s instructions. Luciferase activity was measured in technical triplicate samples, using a TriStar^2^ LB 942 Modular Multimode Microplate Reader (Berthold). Alternatively, cells were harvested in 400 µl of 2x SDS-PAGE sample buffer (0.5 M Tris, pH 6.8, 25% glycerin, 10% SDS, and 0.5% bromophenol blue) and subjected to Western blot analysis using an anti-HA or anti-actin rabbit monoclonal antibody (Abcam), horseradish peroxidase (HRP)-coupled anti-rabbit IgG as secondary antibody (Bio-Rad), and Clarity^TM^ Western ECL substrate (Bio-Rad). Protein quantification was done using ImageLab software (Bio-Rad), and NSs protein levels were normalized to actin levels. GraphPad Prism was used to visualize data and perform statistical analysis. IFN-I promoter inhibition data were analyzed by one-way ANOVA followed by the Bonferroni *post hoc* test, compared to the empty vector control and the wild-type construct. Statistical significance is indicated by asterisks (*) in the graphs: ****p < 0.0001; ***p < 0.001; **p < 0.01; *p < 0.1.

### Immunofluorescence analysis

Vero E6 cells were transfected with 2 μg of the pcDNA3-HA plasmid DNAs encoding the PUUV NSs protein derivatives using Lipofectamine 3000 (Thermo Fisher). For transfection, 2 × 10^5^ cells were seeded simultaneously with transfection mix in a 6-well plate. Forty-eight hours after transfection, the cells were fixed with 4% paraformaldehyde and stained for immunofluorescence analysis using an HA-specific mouse monoclonal antibody (HA-probe antibody (F-7): sc-7392, Santa Cruz Biotechnologies) and an Alexa Fluor 488–labelled anti-mouse secondary antibody (Abcam). Nuclei were stained with 4′,6-diamidino-2-phenylindole (DAPI). For confocal microscopy, cells were seeded on glass cover slips, which were then mounted with Ibidi mounting medium (Ibidi).

### Infection experiments

For Western blot analysis, Vero E6, A549, and MyglaSWRec.B cells were inoculated in a 6-well plate with either PUUV Sotkamo wt or the NSs21Stop strain at an MOI of 0.5 for 1 h at 37 °C in 0.3 ml of medium containing 5% FCS. After adsorption, 1 ml of DMEM with 5% FCS was added, and the cells were incubated at 37 °C for up to 8 days. Cells were harvested at several time points, lysed in 2x SDS-PAGE sample buffer, and subjected to Western blot analysis with the N-protein-specific mouse monoclonal antibody A1C5 (1:500, Promega). A polyclonal rabbit anti-NSs serum (1:50 in PBS-Tween 0.05%) was used for detection of the NSs protein in Vero E6 cells. Primary antibodies were incubated overnight at 4 °C with constant rotation. HRP-labelled goat anti-mouse/anti-rabbit IgG (Bio-Rad) diluted 1:3000 in PBS with 0.05% Tween were used as secondary antibodies for final detection using a ChemiDoc imaging system (Bio-Rad) with development times of 30 min (NSs) or 10 s (N).

For virus growth kinetics, 5 × 10^5^ cells were seeded in a 25-cm^2^ flask the day before inoculation. Cells were incubated with PUUV Sotkamo wt or its NSs21Stop variant at an MOI of 0.1 for 1 h at 37 °C. Thereafter, 6.5 ml of medium was added, and the cells were kept at 37 °C. Supernatants were collected at days 2, 5, and 7 post-inoculation (p.i.). For titration, supernatants of the three cell lines were serially diluted from 10^-1^ to 10^-7^ in DMEM containing 5% FCS in a 96-well plate with three replicates each and processed as described recently [25]. The titers of supernatants of the three cell lines were expressed as the 50% tissue culture infectious dose (TCID_50_)/ml.

## Results

### Expression analysis of hantaviral wild-type NSs-ORFs and inhibition of the IFN-β promoter signaling pathway in human HEK 293-T cells

The S segment of vole-associated orthohantaviruses contains a conserved N-overlapping NSs ORF with three conserved in-frame AUG codons (M1, M14, and M24; shown in yellow in Fig. 1a and b). However, only two of these initiation codons are present in the PHV NSs ORF (M1 and M24) and in the shorter NSs ORFs of SNV and ANDV (M14 and M24), which have their first initiation codon at position M14 (PUUV reference). Of note, the sequences of the KHAV and Rusne virus (RUSV) NSs proteins are longer, with the first AUG located five or four codons ahead of M1 (PUUV reference). In contrast, hantaviruses specific for members of another rodent family (Muridae) or other non-rodent mammals (orders Chiroptera and Eulipotyphla) do not exhibit the coding capacity for an NSs protein due to the lack of translation start codons and to the presence of multiple stop codons (Fig. 1a). Despite the conservation of the start codons in the NSs ORFs of cricetid-borne orthohantaviruses, the coding sequences show varying levels of sequence conservation and variability (Fig. 1b).

**Fig. 2:**
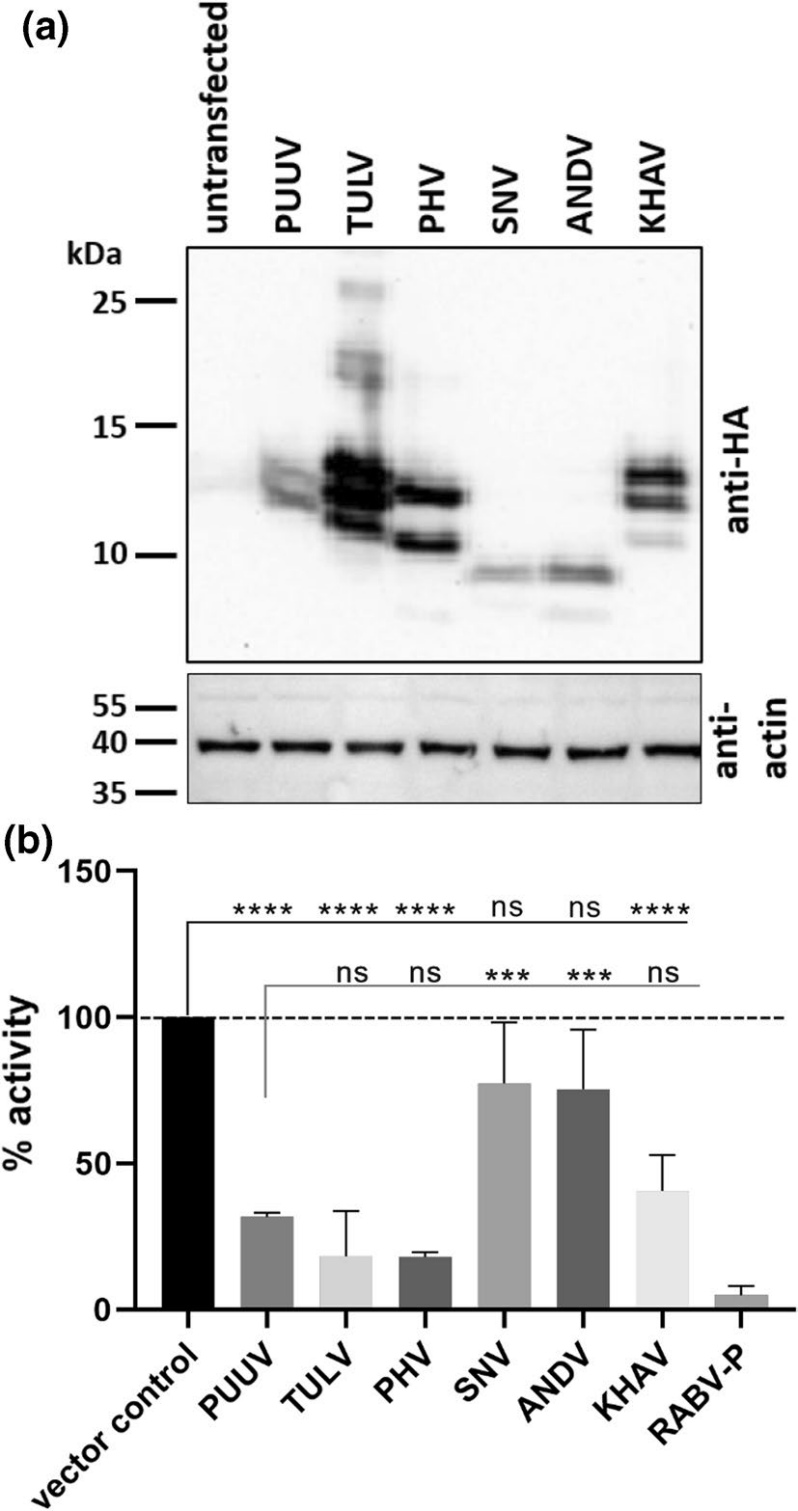
Western blot analysis of expression of the different HA-tagged NSs proteins of hantaviruses in transfected HEK 293-T cells (**a**) and the corresponding results of the RIG-I-activated interferon-β promoter assay (**b**). **a** The different NSs proteins were detected using an anti-HA antibody reacting with their C-terminal HA-tag and the cell lysates were tested using an anti-actin antibody (1:1000) as a protein loading control. For quantification of Western blot results, see Supplementary Figure 2a. **b** HEK 293-T cells were transfected with a plasmid DNA mix of 0.5 µg of p125-FFluc, 0.005 µg of pRluc, 0.5 µg of pcDNA3-huRIG I, and 1 µg of pcDNA3-HA plasmid containing one of the different viral NSs coding sequences. Calculation of % values was done with luciferase luminescence values in reference to the vector control (firefly (FF)/*Renilla* (Rl) value; vector control = 100% activation; vector control without activation = basal level). RABV-P, rabies virus phosphoprotein; PUUV, Puumala virus; TULV, Tula virus; PHV, Prospect Hill virus; SNV, Sin Nombre virus; ANDV, Andes virus; KHAV, Khabarovsk virus. ****, *p* < 0.0001; ns, not significant, as determined by one-way ANOVA followed by the Bonferroni *post hoc* test, compared to the vector control and the PUUV NSs protein

Transfection of HEK 293-T cells with expression plasmids encoding C-terminally HA-tagged NSs proteins from PUUV, TULV, PHV, SNV, ANDV, and KHAV resulted in the detection of one to three protein products (Fig. 2a). All bands in the Western blot assay were detected *via* the C-terminal HA tag, which allows the detection of protein variants that originate from one of the AUG codons near the 5´end of the NSs coding sequence (Fig. 1a, Supplementary Table S1, panel I). NSs proteins of PUUV and PHV gave two distinct bands: the upper band, with a molecular mass around 12 kDa derived from the first 5´ start codon (M1), and the lower band, with a predicted molecular mass around 10.5 kDa for PUUV and 9 kDa for PHV derived from the next downstream AUG codons (M14 and M24, respectively; Figs. 1a and 2a). The KHAV and TULV constructs express three proteins, probably due to translation initiation at each of the three AUG codons within the first 24 codons of their NSs-encoding sequence (Figs. 1a and 2a). As expected, the upper band of KHAV migrated more slowly than those of the other viruses due to a longer full-length NSs (expected molecular mass 12.4 kDa, see Supplementary Table S1). Remarkably, for PUUV, an NSs protein corresponding to translation initiation at codon M24 is not expressed or detectable, in contrast to the NSs expression pattern for KHAV and TULV. The predicted and observed full-length NSs proteins of SNV and ANDV, with an expected molecular mass around 9 kDa, are shorter than those of the vole-associated hantaviruses investigated here (Fig. 1a; Supplementary Table S1, panel I). At least ANDV NSs protein translation seems to be initiated not only at a start codon corresponding to M14 of vole-borne hantavirus NSs but also at a second start codon corresponding to M24 (Figs. 1a and 2a). Of note, TULV NSs protein derivatives are more strongly expressed or more stable than the other NSs proteins, with NSs of PUUV, SNV, and ANDV being more weakly expressed (Supplementary Fig. S2a).

To investigate the influence of orthohantaviral NSs proteins on the IFN-β-promoter-driven induction of IFN-I, we used a dual luciferase reporter assay with RABV-P protein as positive control for inhibition of IFN-I promoter induction. When HEK 293-T cells were transfected with the luciferase reporter constructs and the RIG-I activator plasmid, almost all of the co-transfected orthohantaviral NSs-encoding plasmids led to an efficient reduction of IFN-β promoter activation (Fig. 2b). NSs proteins of PUUV, TULV, PHV, and KHAV strongly inhibited the IFN pathway with 32%, 19%, 18%, and 40% of the IFN-β promoter activity remaining, respectively, as compared to 100% activation observed in the absence of a viral NSs protein (vector control). A potential relationship between the amount of expressed protein and the level of IFN-I promoter inhibition was observed. However, the level of reduction was not significantly different among these NSs proteins from different viruses (Fig. 2 and Supplementary Fig. S2a). PUUV, TULV, PHV, and KHAV NSs proteins are therefore potent inhibitors of this pathway. SNV and ANDV showed 80% of the IFN-β promoter activity induced by RIG-I, but the inhibition was not statistically significant (Fig. 2b). In correlation with these observations, the amount of protein detected was very low (Fig. 2a and Supplementary Fig. S2a).

### Bank vole PUUV field-strain-derived NSs proteins from two endemic regions in Germany exhibit prominent protein expression and interferon antagonist activity

**Fig. 3:**
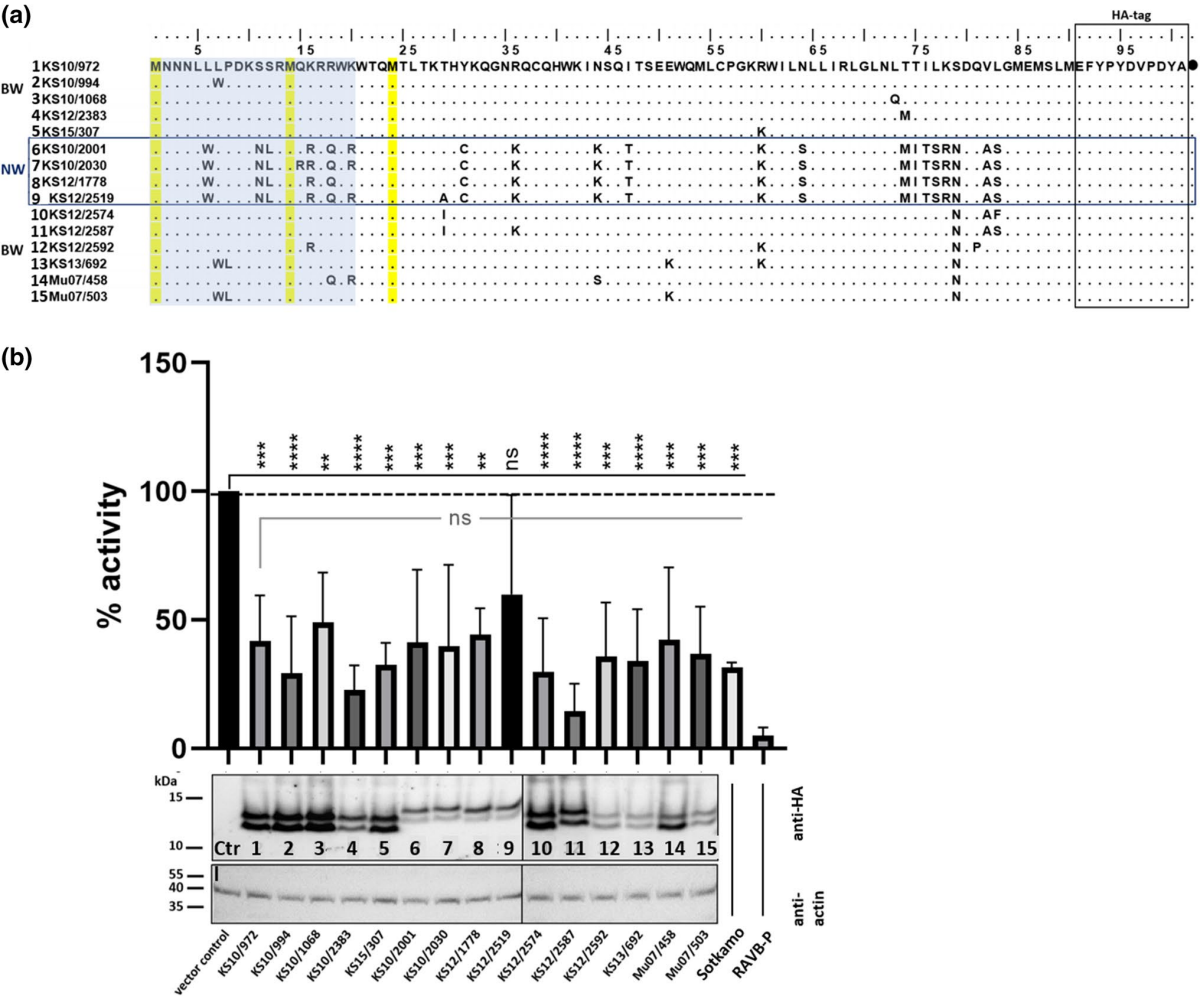
Amino acid sequence comparison of putative NSs proteins of PUUV strains from Baden-Wuerttemberg (BW) and North Rhine-Westphalia (NW) (**a**) and their influence on interferon-β promoter activity and protein expression after RIG-I activation (**b**). **a** Amino acid sequences of PUUV NSs constructs shown with HA tag (see also Supplementary Table S2); (●), stop codon. Methionine (start) codons are highlighted in yellow, and the region of the NSs protein shown to be important for IFN-I inhibition in our analyses is highlighted in blue. **b** Western blot analysis of the expression of the different PUUV NSs constructs and their effect on inhibition of human IFN-β promoter activity. NSs derivatives were expressed by transfection of HEK 293-T cells with 1 µg of plasmid, and the effect of different NSs constructs was measured in the luciferase reporter assay after IFN-β promoter activation by RIG-I. Inhibition of the human IFN-β promoter activity induced by RIG-I was measured in HEK 293-T cells 18 h after transfection with the plasmid DNA mix for the dual luciferase reporter assay and 1 µg of pcDNA3-HA plasmid containing the viral NSs variants. Expression of NSs proteins in cell lysates of the transfected cells was tested in a Western blot assay using an anti-HA rabbit monoclonal antibody and an anti-rabbit HRP-coupled secondary antibody. For quantification of Western blot results, see Supplementary Figure S2b. An anti-actin antibody was used as a loading control. Rabies virus phosphoprotein (RABV-P) was used as a positive control of inhibition. ****p <0.0001; ***p <0.001; **p <0.01; *p <0.1; ns, not significant, as determined by one-way ANOVA followed by the Bonferroni *post hoc* test, compared to the vector control and the KS10/972 construct

The conservation of the PUUV NSs ORF with respect to its position within the S segment and its length of 90 amino acid codons is contrasted by the high amino acid sequence divergence of PUUV strains from different phylogenetic clades (Supplementary Fig. S3) and different endemic regions in Germany (Figs. 1b and 3a). Therefore, we investigated the functional relevance of spatial and temporal NSs sequence variation and the potential consequences of naturally occurring amino acid substitutions within the putative NSs proteins of PUUV strains from bank voles collected in 2007, 2010, 2012, and 2014 at seven trapping sites in BW, southwest Germany, and one site in NW, northwest Germany (Supplementary Table S2). Sequences from the seven different trapping sites in BW had only a few amino acid substitutions (Fig. 3a, numbers 1–5 and 10–15). In contrast, the sequences from NW showed 16.8–24.5% amino acid sequence divergence from the BW sequences (Fig. 3a, 6–9). Expression of the two major NSs variants with translation initiation at M1 and M14 was observed with all of the constructs (Fig. 3b). The NSs proteins from PUUV strains in NW had a higher molecular weight than those from BW (Fig. 3b; NW, lanes 6–9; BW, lanes 1–5 and 10–15; Supplementary Table S1, panel II). All NSs proteins from the different trapping regions showed inhibition of RIG-I-induced IFN-I promoter activation ranging from 14% to 60% of IFN-β promoter activity, which is comparable to the levels observed for PUUV Sotkamo wt NSs, with 32% (Fig. 3b, lanes 1–15). Despite its variation, all NSs variants showed an inhibition of IFN-β promoter activity that is not significantly different from that of KS10/972. There was no obvious correlation between increased or decreased IFN-I promoter inhibition and a specific amino acid exchange, and there was no obvious correlation with the amount of protein (see also protein quantification in Supplementary Fig. S2b).

### Importance of the N-terminal region of the PUUV NSs protein for its inhibitory effect on IFN-I promoter induction

**Fig. 4:**
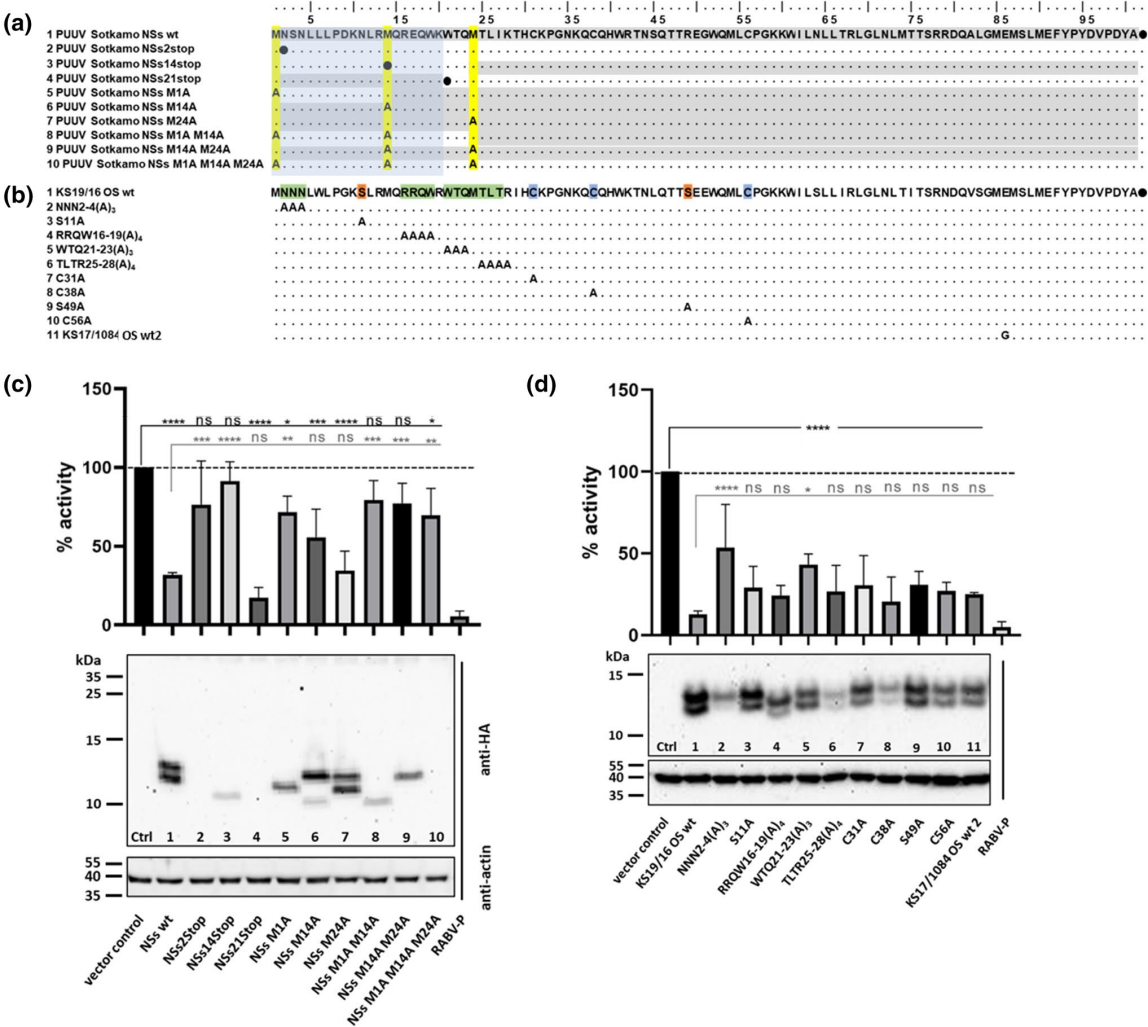
Influence of mutations in the Puumala virus (PUUV) NSs ORF on interferon-β promoter activity and protein expression after RIG-I activation. **a** Amino acid sequences of PUUV Sotkamo NSs constructs shown with an HA tag. Conserved putative start (methionine) codons are highlighted in yellow. Detected protein variants are shown in grey from the first potential start codon in the different constructs with a stop codon (●) or alanine substitution of the different methionine residues M1, M14, and M24. **b** Amino acid exchanges introduced at particular putative functional residues in NSs of PUUV OS strain KS19/16, with cysteine, serine, and hydrophobic regions, highlighted in blue, red, and green, respectively. **a**, **b** The region of NSs shown to be important for IFN-I promoter inhibition in our analyses is highlighted in blue. **c** Western blot analysis of the expression of the different PUUV NSs constructs shown in panel a and their effect on inhibition of human IFN-β promoter activity. NSs derivatives were expressed by transfection of HEK 293-T cells with 1 µg of plasmid, and the effect of alternative start codon usage of NSs constructs was measured in the luciferase reporter assay after RIG-I-mediated IFN-β activation by the same plasmid mix used in the experiment shown in Fig. 2b. **d** Influence of alanine codon mutagenesis in the NSs protein of PUUV strain KS19/16 OS wt from Osnabrück (OS; see panel b). Inhibition of the RIG-I-induced human IFN-β promoter activity was measured in HEK 293-T cells 18 h after transfection with the plasmid DNA mix for the dual luciferase reporter assay and 1 µg pcDNA3-HA plasmid containing one of the viral NSs coding sequences. Expression of NSs proteins in cell lysates of the transfected cells was tested in a Western blot assay using an anti-HA rabbit monoclonal antibody and an anti-rabbit HRP-coupled secondary antibody. For quantification of Western blot results, see Supplementary Figure S2c. An anti-actin antibody was used as a loading control. Rabies virus phosphoprotein (RABV-P) was used as a positive control of inhibition and is shown in the last lane in panels c and d. ****p < 0.0001; ***p < 0.001; **p < 0.01; *p < 0.1; ns, not significant, as determined by one-way ANOVA followed by the Bonferroni *post hoc* test, compared to the vector control or wt constructs

For further characterization of the PUUV NSs protein and investigation of the importance of leaky scanning in generating NSs variants, eight mutants of the PUUV Sotkamo wt NSs sequence were generated by introduction of stop codons or by alanine codon substitution of methionine codons, and combinations of these mutations were also generated (Fig. 4a, Supplementary Table S1, panel III). The expression patterns of these mutants were analyzed by Western blot assay, and their inhibitory function in HEK 293-T cells was investigated using a RIG-I-activated IFN-β luciferase reporter assay. Expression of the C-terminally-HA tagged protein variants after transfection of Vero E6 cells was confirmed by immunofluorescence assay (Supplementary Fig. S1). When a stop codon was introduced at codon position 2 (NSs2Stop; Fig. 4a), no protein could be detected, and the inhibitory effect of the PUUV NSs was not significant (76% remaining activity) when compared to the empty vector control (Fig. 4c, lane 2 and Supplementary Fig. S1). This indicated that the NSs protein, and not the RNA, was responsible for the inhibitory effects on the IFN-I induction, as the mRNA should be transcribed in the same way for both constructs (NSs wt and NSs2Stop). The NSs14Stop variant expressed a protein of around 8.9 kDa that was translated from the third start codon at position 24. This shortened NSs protein also showed no significant inhibitory effect (91% activity remaining) compared to the control in the reporter assay (Fig. 4c, lane 3). Similar to what was seen by Western blot analysis, a weaker signal of immunofluorescence staining was obtained with the NSs14Stop-derived protein when compared to the wt NSs protein (Supplementary Fig. S1). Reduced immunofluorescence and Western blot signals were also observed for the M1A M14A double mutant, which lacks the first two start codons and therefore expresses the same NSs protein variant of 8.9 kDa starting at codon 24 as mutant NSs14Stop (Fig. 4c, lane 8, Supplementary Fig. S1). The double mutant M1AM14A showed no significant inhibition (79% activity) of the IFN-β promoter activity (Fig. 4c, lane 8) compared to the vector control, as was the case for the NSs14Stop construct (lane 3). In contrast, the transfected NSs21Stop plasmid construct (NSs-lacking virus variant) showed a very strong effect (17% activity) on IFN-I promoter inhibition compared to the wt (32% activity), although no HA-tagged protein could be detected (Fig. 4c, lane 4). Of note, the HA-tag coding sequence in this construct is located downstream of the entire NSs coding sequence, and therefore, peptides initiating at codons 1 or 14 and terminating at codon 21 could not be detected due to a missing tag at the C-terminus of these short peptides. The absence of any NSs protein product for the NSs21Stop construct, including the putative amino acid 24-90 protein, was shown by Western blot analysis (Fig. 4c, lane 4) and immunofluorescence analysis (Supplementary Fig. S1). In contrast, reinitiation at start codon M24 was observed for the NSs14Stop, M14A, and M1AM14A constructs, but the expression level was low (Fig. 4c, lanes 3, 6 and 8). The fusion of the NSs1-20 peptide with a C-terminal HA tag or the FMDV-1A protein as a C-terminal tag resulted in a loss of inhibitory activity (data not shown).

Substitution of the first methionine start codon by alanine codon (M1A) in the NSs sequence of PUUV resulted in the expression of only one protein variant corresponding to the shorter NSs of the two distinct bands found with wt NSs (Fig. 4c, lane 5). This pattern is most likely due to translation initiation at the M14 AUG codon. This was accompanied by a highly reduced inhibitory effect on IFN-β promoter induction (Fig. 4c), confirming the importance of the N-terminal region of the NSs protein for its function in blocking IFN-I signaling. When the second putative start codon was substituted (M14A), the full-length NSs was expressed together with a smaller band (8.9 kDa) of weaker intensity (Fig. 4c, lane 6), which was also observed for the NSs14Stop mutant (Fig. 4c, lane 3), but not with the wt NSs (Fig. 4c, lane 1). This smaller NSs variant probably arose from translation initiation at codon M24. Only an activity of 56% of IFN-I promoter induction was observed with this mutant M14A protein as compared to the wt NSs protein. Alanine codon substitution of the third AUG (M24A) resulted in detection of the full-length NSs band together with the 10.3-kDa band representing the same expression pattern as observed with the wt NSs ORF (Fig. 4c, compare lanes 7 and 1). This mutation resulted in 34% promoter activity, an inhibition comparable to that of the wt NSs with 32% activity, as expected from the expression pattern. However, NSs proteins with combinations of these alanine/start codon substitutions had no inhibitory effect in the luciferase reporter assay (Fig. 4c, lanes 8-9). The double mutant M14A M24A, although expressing the expected single band of full-length PUUV NSs protein, induced nonsignificant background reduction, with 77% of the IFN-β promoter activity. Therefore, these two substitutions within the first 24 amino acids had a strong impact on the inhibitory function of NSs. In the case of the triple mutant M1A M14A M24A, no protein could be detected (Fig. 4b, lane 10), and as expected, only a minor influence on IFN-I signaling could be observed.

### Mutational analysis of NSs proteins from PUUV field strains from the Osnabrück region confirmed the phenotypic plasticity of the NSs protein

For identification of important amino acid residues or motifs in the PUUV NSs protein responsible for its IFN-I inhibitory function, we replaced amino acid residues in conserved regions (Fig. 4b) of the NSs protein of PUUV OS strain KS19/16 by alanine. This field strain NSs sequence (Fig. 4d, lane 1, 13% of IFN-β promoter activity) as well as that of another field strain, PUUV OS KS17/1084 (Fig. 4d, lane 11, 25% of IFN-β promoter activity) showed higher IFN-I inhibition levels than the laboratory-adapted PUUV Sotkamo wt-derived NSs sequence (Fig. 2b, 32% of IFN-β promoter activity). The NSs sequence of strain KS19/16 was therefore selected for the mutagenesis studies, as mutations affecting the inhibitory activity would be better recognized using this strain. We targeted the N-terminal NNN stretch (residues 2-4), serine S11A, and S49A, as they constitute potential phosphorylation sites, cysteine residues C31A, C38A, and C56A for the formation of disulfide bonds or catalytic functions, and larger hydrophobic regions of interest, RRQW16-19(A)_4_ and TLTR25-28(A)_4_, by site directed mutagenesis (Fig. 4b). Most of the alanine replacement variants showed a clear inhibition of the IFN-β promoter activity, ranging from 13% to 31% activity compared to 100% without NSs protein. Only the N-terminal amino acid exchanges NNN2-4(A)_3_ (Fig. 4d, lane 2, 54%) and WTQ21-23(A)_3_ (lane 5, 43%) resulted in reduced IFN-I inhibition capacity that was significantly different from that of the parental KS19/16 wt NSs protein (Fig. 4d, lane 1). Western blot analysis showed that all NSs-HA constructs were strongly expressed in transfected HEK 293-T cells, ranging from 50%, 70%, or 80% to 100% of the expression level of the KS 19/16 wt protein (Supplementary Fig. S2c). The constructs produced double bands about 1 kDa apart from each other (Fig. 4d), corresponding to the two bands seen with the wt NSs protein of the PUUV Sotkamo strain, which is thought to initiate at the M1 and M14 start codons (Supplementary Table S1, panel IV). However, the amount of NSs-HA-tagged protein appeared to be different for all variants. There was no obvious correlation between the amount of NSs protein present in the assay and the level of the inhibitory effect on IFN-I promoter induction. Thus, an influence on NSs inhibitory function can be observed for the tested mutants, but no essential residue or motif could be identified, except at residues 2-4, indicating a high phenotypic plasticity of the NSs protein.

### Replication of PUUV in different cell lines is not affected by NSs protein variants

**Fig. 5:**
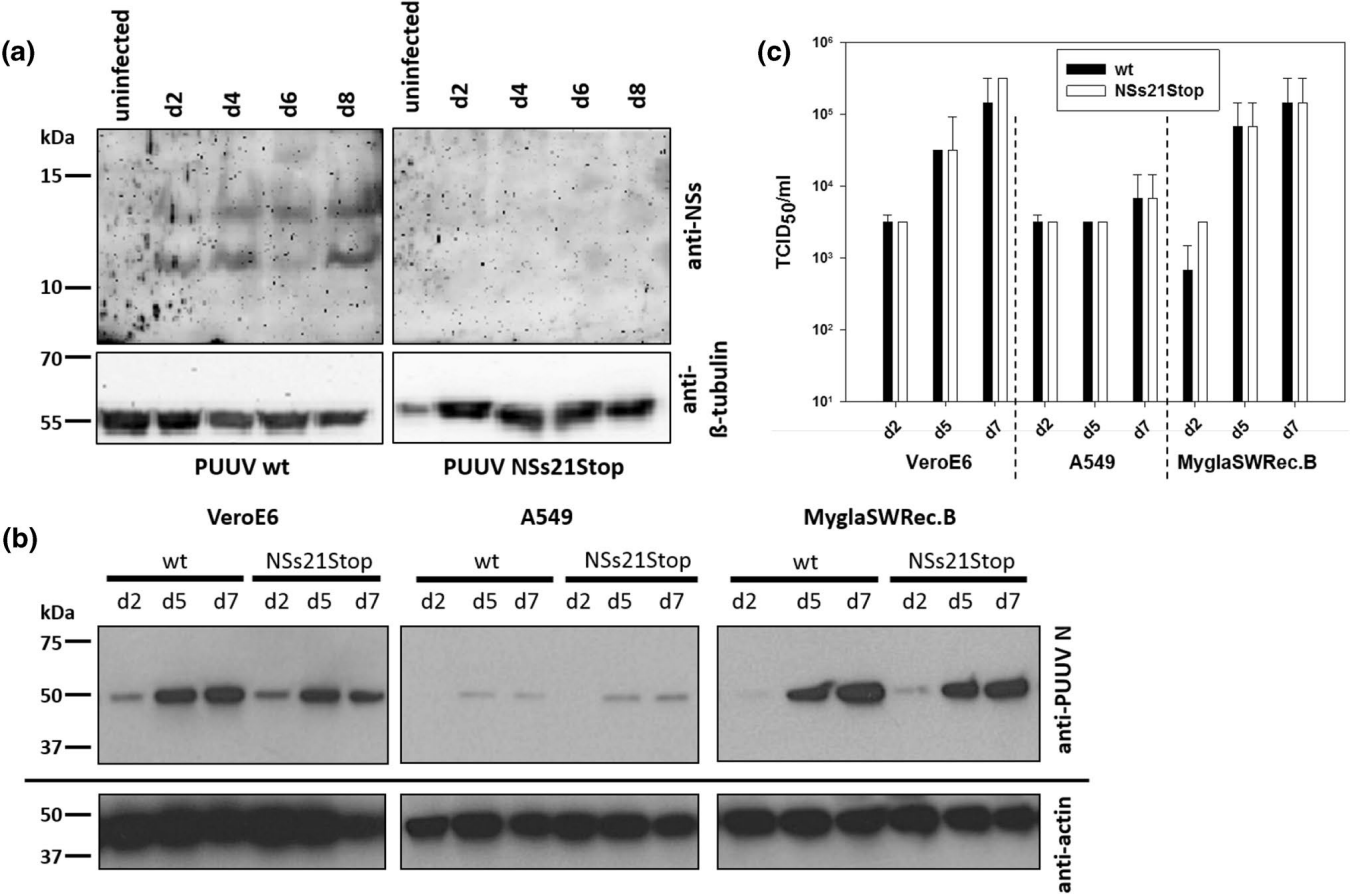
Expression of PUUV Sotkamo NSs protein after infection of Vero E6 cells (**a**) and replication of PUUV Sotkamo wt and NSs21Stop strains in Vero E6, A549, and MyglaSWRec.B cells analysed by Western blot assay (**b**) and virus titration (**c**). Cells were infected at an MOI of 0.5 and harvested at the indicated time points. For Western blot analysis of NSs protein, a polyclonal rabbit anti-NSs serum (1:50 in PBS with 0.05%Tween) was incubated overnight at 4 °C. The N protein was detected using the specific mouse monoclonal antibody A1C5 (1:500, Promega). Titers of supernatants of the three cell lines are expressed as the 50% tissue culture infectious dose (TCID_50_)/ml determined by titration on Vero E6 cells by indirect immunofluorescence assay for the PUUV N protein. Calculation was done by the Spearman/Kärber method, and the mean titers of three replicates each are shown

First, we analyzed NSs expression of PUUV Sotkamo in *in vitro*-infected cells using a rabbit anti-PUUV NSs serum. The PUUV NSs protein could be detected from day 2 to day 8 p.i. in Vero E6 cells infected at an MOI of 0.1 (Fig. 5a, left panel). Interestingly, two bands could be detected by Western blot analysis, suggesting that the first two AUG codons (Figs. 1a and 5a, left panel) in the NSs ORF are used as alternative starting points for leaky scanning translation of the NSs protein. When we used the PUUV Sotkamo NSs variant virus (NSs21Stop) [19], no specific protein was detected using the anti-NSs serum (Fig. 5a, right panel).

To evaluate the influence of the NSs protein on viral replication during infection, we infected three different cell lines with wt PUUV Sotkamo and the PUUV Sotkamo strain with a truncated NSs (NSs21Stop). Vero E6 cells (African green monkey kidney cells), an IFN-I deficient reference cell line, human A549 cells, and bank vole renal epithelial cells (MyglaSWRec.B) were simultaneously infected at an MOI of 0.5 and assessed for their expression of N protein and titer production as evidence of PUUV replication. No obvious differences in the level of N expression between the two virus isolates were seen in any of the cell lines by Western blot analysis. However, Vero E6 and MyglaSWRec.B cells showed high expression levels of PUUV N on days 5 and 7 p.i., whereas in A549 cells, lower expression was observed for both viruses at all time points (Fig. 5b). A similar observation was made for the virus titers in the supernatants of all three cell lines (Fig. 5c). The amount of virus in the supernatant increased from day 2 to day 7 in Vero E6 and MyglaSWRec.B cells from 10^3^ to 10^5^ TCID_50_/ml without differences between the wt and mutant PUUV strain. A549 cells showed only a weak increase in viral titer, which is in line with the observations from Western blot analysis (Fig. 5b and c).

## Discussion

Our transfection studies indicated the synthesis of different NSs protein variants of various cricetid-borne orthohantaviruses that can be explained by translation initiation at multiple start codons, M1, M14, or M24, by a leaky scanning mechanism. These three putative start codons were found to be conserved in NSs ORFs of all PUUV strains analyzed. A leaky-scanning mechanism for NSs translation initiation in which the upstream AUG of the N protein is bypassed was shown for ANDV S mRNA, but without detection of different NSs protein variants [5]. This was confirmed to occur during infection with PUUV strain Sotkamo, as two NSs protein bands, although in low amounts, were detected by a newly generated anti-NSs serum in the lysates of PUUV-wt-infected cells. Mutational analysis of the potential start codons in the transfection system gave additional proof for the use of different NSs initiation codons. Alanine substitution at the first start codon resulted in only one NSs variant, starting at position M14, whereas the wt showed two proteins, starting at M1 and M14. Therefore, in these two cases (wt and M1A), no translation initiation by leaky scanning occurred at the third start codon (M24). However, initiation at codon 24 was observed for the NSs variants M14A, M1A M14A, and NSs14Stop, probably because of the substitution of the AUG at position 14, which is located in a favorable Kozak sequence context [26], allowing translational scanning to continue to the next AUG at position 24.

The lack of activity of the full-length NSs protein alone (construct M14A M24A) and the protein starting from M24 alone (constructs M1A M14A and NSs14Stop) raises the question whether two (or three) NSs protein variants are needed for a fine-tuned downregulation of IFN-I production mediated by their interaction (NSs amino acid residues 1–90 and 14–90) or an alternative influence of smaller NSs ORF products. This conclusion is supported by the observation that mutants in which two of the three AUG codons are mutated did not show any inhibitory function in our luciferase system. The almost complete loss of IFN-I inhibitory function when translation of any NSs protein product seems to be blocked by a stop codon at position 2 indicates that the protein itself causes the inhibitory effects on IFN-I induction, as the encoding mRNAs of all constructs are almost identical except for the few introduced single nucleotide exchanges. The block of translation through insertion of a stop codon directly downstream of the first start codon apparently prevents scanning beyond this site to the next start codon, or the amount of protein expressed is too low to be detected. The same might be true for the stop codon at position 21; the proximity of this stop codon to the next start codon (M24) might have resulted in a lack of leaky scanning and the absence of NSs 24-90 in the transfection experiment with NSs21Stop and the infection experiment with the corresponding PUUV strain.

Western blot and IFN-promoter-driven luciferase reporter assays both showed that NSs proteins from 17 bank vole field strains from three endemic regions in Germany reduced the IFN-β promoter activity significantly, with 14–60% of the activity remaining when compared to the vector control. These results were comparable to the 32% observed with the NSs protein of the cell-culture-adapted PUUV Sotkamo wt strain. Additional amino acid exchanges introduced into the PUUV NSs protein of a bank vole field strain showed 21–54% promoter activity, compared to 13% for the unmutated wt, and therefore did not interfere significantly with protein function. Furthermore, the substitution of potentially functional amino acid residues, such as cysteine or serine, showed 21–31% activity and therefore did not have an effect on the inhibitory function of the NSs protein. Only the exchange of three or four amino acid residues in a row significantly reduced the inhibitory effect of NSs, but only to a small extent, with 54% and 43% IFN-β promoter activity, compared to 13% with the wt construct. Interestingly, the NNN(2-4)AAA exchange resulted in a significant reduction of inhibitory activity. This phenomenon might be related to the loss of the methionine at the N-terminus, which is most likely cleaved because of the changed second amino acid of the NSs protein (N to A) [27], but the lower level of expression of the full-length NSs protein might also play a role. The functional activity of the different wt NSs proteins suggests a high phenotypic plasticity of the NSs protein, showing that it tolerates the exchange of amino acid residues.

PUUV and TULV NSs proteins have already been shown to be expressed during infection and to inhibit NF-κB- and IRF-3-responsive promoters after poly(I:C) stimulation [6]. A recent study also showed that the ANDV NSs protein antagonizes the IFN-I response by inhibiting IFN-I signaling downstream of RIG-I and MDA-5 but upstream of TBK-1 [28]. We expressed hantaviral NSs ORFs from PUUV, TULV, PHV, and KHAV, and they all inhibited IFN-I induction. Otarola et al. showed antagonistic effects on IFN-I induction of ANDV NSs protein that reduced IFN-I signaling to about 50% [28]. In our experiments, expression of the NSs proteins of ANDV and the related SNV did not show a significant effect on IFN-I signaling. This is mainly due to the low level of protein expression observed here. This can be explained by the use of different expression constructs, as we used a C-terminal HA tag and not an N-terminally tagged NSs protein, which could be more stable. The different transfection setups in general can also cause these differences in inhibition potency. However, in our experiments, comparison to other NSs constructs, all with C-terminal HA-tag, still showed a higher potency of IFN-I promoter inhibition by the NSs proteins of PUUV, TULV, PHV, and KHAV than by that of ANDV (and the related SNV). The expression patterns of the NSs proteins of the different virus strains used here were consistent with the multiple internal start codons for each viral NSs, suggesting selection pressure for the conservation of multiple start codons within the NSs ORF of various orthohantaviruses (see Figs. 1 and 3a and Supplementary Fig. S3).

The N-terminal region of the NSs protein appeared to have a considerable effect on IFN-β promoter activity. The smallest (10 kDa) variant of PUUV NSs, expressed by the M1A M14A double mutant or the NSs14Stop construct, showed no significant reduction in IFN-I induction, but this could be linked to the low level of expression of this fragment. In contrast, the NSs21Stop construct, encoding only the first 20 N-terminal amino acids of the NSs protein, had the highest inhibitory effect. This points to a role of the first 20 amino acids of NSs in the inhibition of IFN-I induction. In a previous study, the same fragment with an N-terminal HA-tag showed only a marginal inhibitory effect [8]. Therefore, we controlled the activity of the NSs1-20 by C-terminal fusion of an HA tag or a protein of FMDV that does not show inhibition in the IFN-I signaling assay on its own. The lack of inhibitory activity of these NSs1-20-HA-tag and NSs1-20-FMDV-1A-tag proteins is consistent with the results of the previous study and suggests a high vulnerability of the activity of the short segment of the NSs protein to the addition of another protein. This seems to be consistent with the effect of the NNN2-4AAA substitutions on the inhibitory activity of the entire NSs protein. Similarly, N-terminally truncated variants of NSs from BUNV were still able to interact with Med8, a factor involved in regulating the activity of cellular RNA polymerase II, but failed to degrade cellular RNA polymerase II and thereby block IFN transcription [29]. Recently, the inhibition of IFN β induction by a 22-amino-acid-long peptide within the ORF3b protein of SARS-CoV-2, generated by a premature stop codon, has been described [30].

Despite the special importance of the N-terminal region of PUUV NSs protein, the rest of the protein also seems to be important for IFN β inhibition: The HA-tagged NSs24-90 protein synthesized in a transfection system inhibits the IFN promoter induction, but not at the same level as the corresponding complete NSs protein [8]. The conservation of the length and position of the NSs ORF in most arvicoline-associated orthohantaviruses additionally suggests the importance of the entire NSs protein. Interestingly, the N-terminally elongated NSs proteins of KHAV and RUSV lack the start codon at position 1 but instead have a start codon at position -5 or -4, thereby conserving the start codons at three positions of the NSs ORF. Thus, NSs might have several regions that are important for IFN-I inhibition, one at the N-terminus and another in the remaining portion of the protein, each able on its own to act on interferon signaling, and different NSs proteins generated simultaneously via leaky scanning may act synergistically. The observed inhibitory function of each NSs protein variant might be influenced by its expression level and the effects of mutations on protein secondary structure and stability.

In line with these findings from transfection experiments, the NSs21Stop variant of PUUV strain Sotkamo replicates in different cell lines to the same extent as the wt counterpart of this PUUV strain. The similar level of replication of both PUUV strains in each of the three different cell lines shows that the truncated NSs might be sufficient to fulfill the function of interferon inhibition in absence of the full-length protein. Interestingly, a comparison of wt and NSsStop strains of PUUV and TULV in A549 cells indicated that the strains with an intact NSs ORF, but not the strains with a truncated NSs ORF, induced IFN I and IFN-induced genes at 5 days postinfection [8]. Taken together, these findings may suggest multiple functions of NSs and its putative domains, a role of other viral proteins, and additional pathways modulated by orthohantaviruses.

In conclusion, the NSs ORF of cricetid-borne hantaviruses is expressed as different variants via leaky scanning, and the expressed NSs proteins inhibit IFN-I promoter activity. The NSs protein of PUUV demonstrated a high phenotypic plasticity, as shown by the activity of natural NSs proteins with highly divergent amino acid sequences and *in vitro-*modified NSs variants. The N-terminal part of the PUUV NSs protein seems to be important for its function, but the amino acid 1-20 peptide is highly vulnerable to addition of tags. The high conservation of the 90-codon-long NSs ORF in field strains confirms an additional functional role of the remaining part of the NSs protein. The manipulation of the potential translation initiation sites has a much stronger influence on NSs activity. The assumption that different NSs ORF products could interact for precise function remains speculative and needs to be investigated. Future investigations should confirm the results with other pathogenic and low- or non-pathogenic hantaviruses, identify essential targets for NSs function, and investigate potential interactions of different NSs translation products and the involvement of other viral proteins and their cellular interaction partners. Finally, a virus-reservoir cell system would be of substantial interest to understand the influence of hantaviruses on the immune system of their reservoir hosts.

## Supplementary Information

Below is the link to the electronic supplementary material.

Supplementary file1 (DOCX 1167 KB)

## References

[1] Meyer BJ, Schmaljohn CS (2000). Persistent hantavirus infections: characteristics and mechanisms. Trends Microbiol.

[2] Vaheri A, Henttonen H, Voutilainen L, Mustonen J, Sironen T, Vapalahti O (2013). Hantavirus infections in Europe and their impact on public health. Rev Med Virol.

[3] Hedil M, Kormelink R (2016). Viral RNA silencing suppression: The enigma of bunyavirus NSs proteins. Viruses.

[4] Plyusnin A (2002). Genetics of hantaviruses: implications to taxonomy. Adv Virol.

[5] Vera-Otarola J, Solis L, Soto-Rifo R, Ricci EP, Pino K, Tischler ND, Ohlmann T, Darlix JL, Lopez-Lastra M (2012). The Andes hantavirus NSs protein is expressed from the viral small mRNA by a leaky scanning mechanism. J Virol.

[6] Jääskelainen KM, Kaukinen P, Minskaya ES, Plyusnina A, Vapalahti O, Elliott RM, Weber F, Vaheri A, Plyusnin A (2007). Tula and Puumala hantavirus NSs ORFs are functional and the products inhibit activation of the interferon-beta promoter. J Med Virol.

[7] Virtanen JO, Jääskelainen KM, Djupsjobacka J, Vaheri A, Plyusnin A (2010). Tula hantavirus NSs protein accumulates in the perinuclear area in infected and transfected cells. Adv Virol.

[8] Gallo G, Caignard G, Badonnel K, Chevreux G, Terrier S, Szemiel A, Roman-Sosa G, Binder F, Gu Q, Da Silva Filipe A, Ulrich RG, Kohl A, Vitour D, Tordo N, Ermonval M (2021). Interactions of viral proteins from pathogenic and low or non-pathogenic orthohantaviruses with human type I interferon signaling. Viruses.

[9] Billecocq A, Spiegel M, Vialat P, Kohl A, Weber F, Bouloy M, Haller O (2004). NSs protein of Rift Valley fever virus blocks interferon production by inhibiting host gene transcription. J Virol.

[10] Schoen A, Weber F (2015). Orthobunyaviruses and innate immunity induction: alieNSs vs PredatoRRS. Eur J Cell Biol.

[11] Habjan M, Pichlmair A, Elliott RM, Overby AK, Glatter T, Gstaiger M, Superti-Furga G, Unger H, Weber F (2009). NSs protein of Rift Valley fever virus induces the specific degradation of the double-stranded RNA-dependent protein kinase. J Virol.

[12] Hollidge BS, Weiss SR, Soldan SS (2011). The role of interferon antagonist, non-structural proteins in the pathogenesis and emergence of arboviruses. Viruses.

[13] Kalveram B, Ikegami T (2013). Toscana virus NSs protein promotes degradation of double-stranded RNA-dependent protein kinase. J Virol.

[14] Thomas D, Blakqori G, Wagner V, Banholzer M, Kessler N, Elliott RM, Haller O, Weber F (2004). Inhibition of RNA polymerase II phosphorylation by a viral interferon antagonist. J Biol Chem.

[15] Weber F, Bridgen A, Fazakerley JK, Streitenfeld H, Kessler N, Randall RE, Elliott RM (2002). Bunyamwera bunyavirus nonstructural protein NSs counteracts the induction of alpha/beta interferon. J Virol.

[16] Kell AM, Gale M (2015). RIG-I in RNA virus recognition. Virology.

[17] Basler CF, Garcia-Sastre A (2002). Viruses and the type I interferon antiviral system: induction and evasion. Int Rev Immunol.

[18] Haller O, Staeheli P, Schwemmle M, Kochs G (2015). Mx GTPases: dynamin-like antiviral machines of innate immunity. Trends Microbiol.

[19] Rang A, Heider H, Ulrich R, Krüger DH (2006). A novel method for cloning of non-cytolytic viruses. J Virol Methods.

[20] Castel G, Couteaudier M, Sauvage F, Pons JB, Murri S, Plyusnina A (2015). Complete genome and phylogeny of Puumala hantavirus isolates circulating in France. Viruses.

[21] R Core team (2015) R: A Language and Environment for Statistical Computing. R Foundation for Statistical Computing

[22] Strandin T, Smura T, Ahola P, Aaltonen K, Sironen T, Hepojoki J, Eckerle I, Ulrich RG, Vapalahti O, Kipar A, Forbes KM (2020). Orthohantavirus isolated in reservoir host cells displays minimal genetic changes and retains wild-type infection properties. Viruses.

[23] Brune KD, Leneghan DB, Brian IJ, Ishizuka AS, Bachmann MF, Draper SJ, Biswas S, Howarth M (2016). Plug-and-display: decoration of virus-like particles via isopeptide bonds for modular immunization. Sci Rep.

[24] Brzózka K, Finke S, Conzelmann KK (2005). Identification of the rabies virus alpha/beta interferon antagonist: phosphoprotein P interferes with phosphorylation of interferon regulatory factor 3. J Virol.

[25] Binder F, Lenk M, Weber S, Stoek F, Dill V, Reiche S, Riebe R, Wernike K, Hoffmann D, Ziegler U, Adler H, Essbauer S, Ulrich RG (2019). Common vole (*Microtus arvalis*) and bank vole (*Myodes glareolus*) derived permanent cell lines differ in their susceptibility and replication kinetics of animal and zoonotic viruses. J Virol Methods.

[26] Kozak M (1989). Context effects and inefficient initiation at non-AUG codons in eucaryotic cell-free translation systems. Mol Cell Biol.

[27] Sherman F, Stewart JW, Tsunasawa S (1985). Methionine or not methionine at the beginning of a protein. BioEssays.

[28] Otarola JV, Solis L, Lowy F, Olguin V, Angulo J, Pino K, Tischler ND, Otth C, Padula P, Lopez-Lastra M (2020). The Andes orthohantavirus NSs protein antagonizes the type I interferon response by inhibiting MAVS signaling. J Virol.

[29] van Knippenberg I, Carlton-Smith C, Elliott RM (2010). The N-terminus of Bunyamwera orthobunyavirus NSs protein is essential for interferon antagonism. J Gen Virol.

[30] Konno Y, Kimura I, Uriu K, Fukushi M, Irie T, Koyanagi Y, Sauter D, Gifford RJ, Nakagawa  S, Sato K, USFQ-COVID19 Consortium (2020). SARS-CoV-2 ORF3b Is a Potent Interferon Antagonist Whose Activity Is Increased by a Naturally Occurring Elongation Variant. Cell Rep.

[31] Binder F, Ryll R, Drewes S, Jagdmann S, Reil D, Hiltbrunner M, Rosenfeld UM, Imholt C, Jacob J, Heckel G, Ulrich RG (2020). Spatial and Temporal Evolutionary Patterns in Puumala Orthohantavirus (PUUV) S Segment. Pathogens.

