# Supplementary material for: Inhibition of interferon I induction by non-structural protein NSs of Puumala virus and other vole-associated orthohantaviruses: phenotypic plasticity of the protein and potential functional domains
Source: Arch Virol. 2021 Aug 13;166(11):2999–3012. doi: 10.1007/s00705-021-05159-y (PMC8362652; doi:10.1007/s00705-021-05159-y)
Supplement: Supplementary file 1 — Supplementary file1 (DOCX 1167 KB) [file 705_2021_5159_MOESM1_ESM.docx]

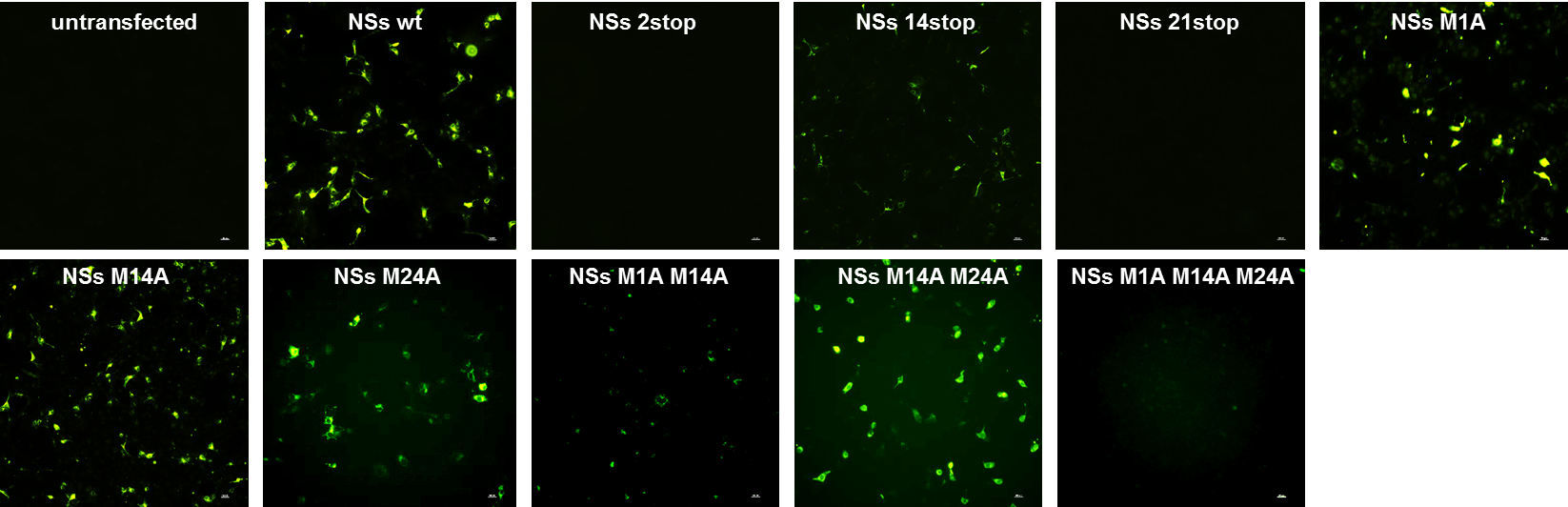


**Supplementary Figure 1: Immunofluorescence analysis of PUUV Sotkamo NSs expression constructs.** VeroE6 cells were transfected with 2µg plasmid DNA encoding the respective HA-tagged PUUV NSs protein. Cells were seeded simultaneously with transfection mix in a 6-well plate. 48 h after transfection, cells were stained for immunofluorescence analysis using an anti-HA specific monoclonal antibody and an Alexa fluor 488 labelled secondary antibody (green fluorescence).


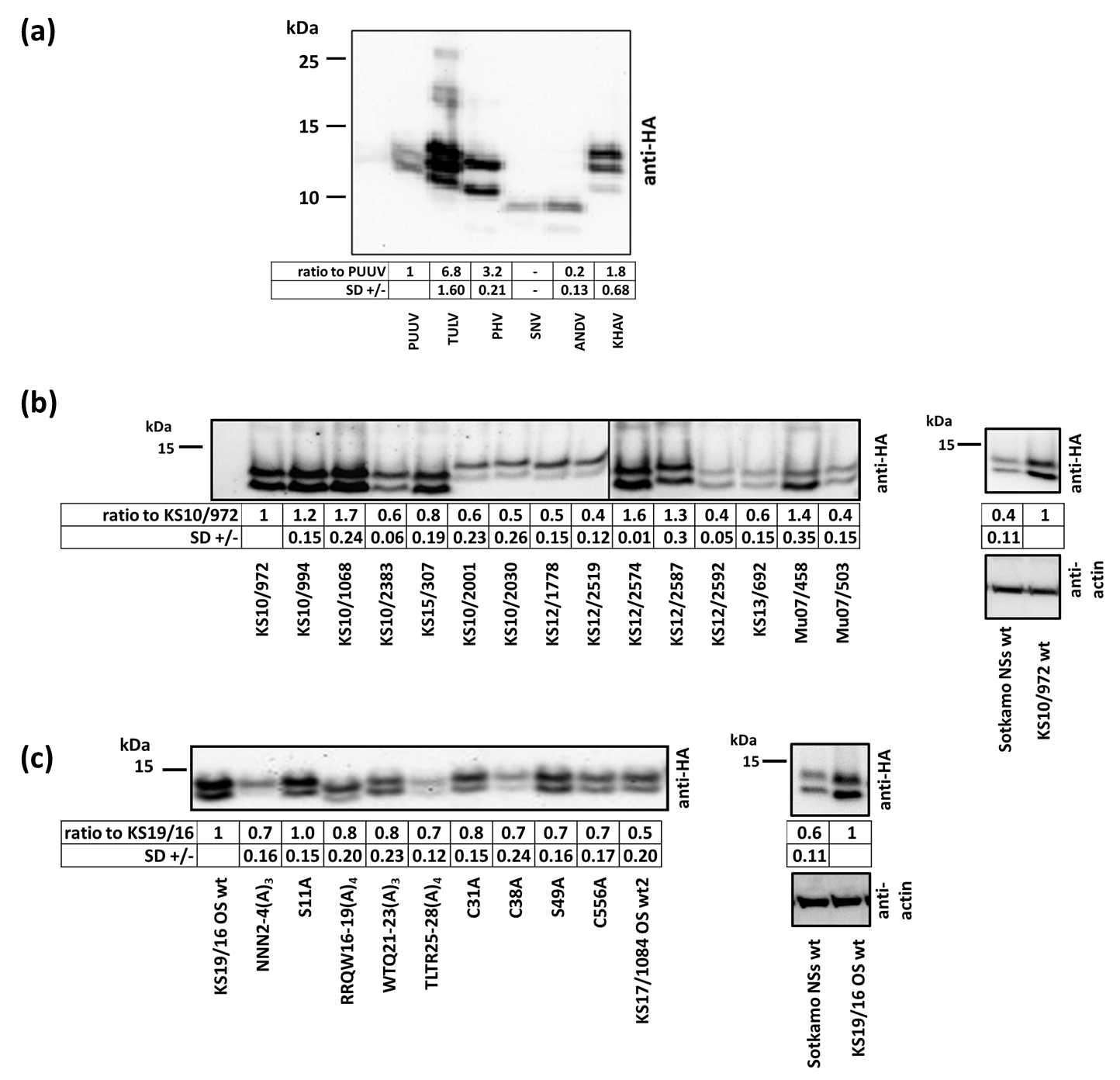


**Supplementary Figure 2: Quantification of NSs protein expression in Western blot assays shown in Figures 2a, 3b and 4d.** (a) Quantification of different orthohantavirus NSs proteins detected in Figure 2a, (b) NSs proteins of regional PUUV field strains detected in Figure 3b, and (c) alanine mutagenesis of PUUV NSs proteins shown in Figure 4d. Values are given as ratio to the corresponding wild-type protein and standard deviations (SD) were calculated. Values for SNV in (a) could not be calculated because protein levels being too low for quantification analysis. NSs protein expression of regional PUUV wild-type strains from Baden-Wuerttemberg, North Rhine-Westphalia and Osnabrück (OS) was compared to the PUUV Sotkamo wild-type NSs protein in b and c.

**
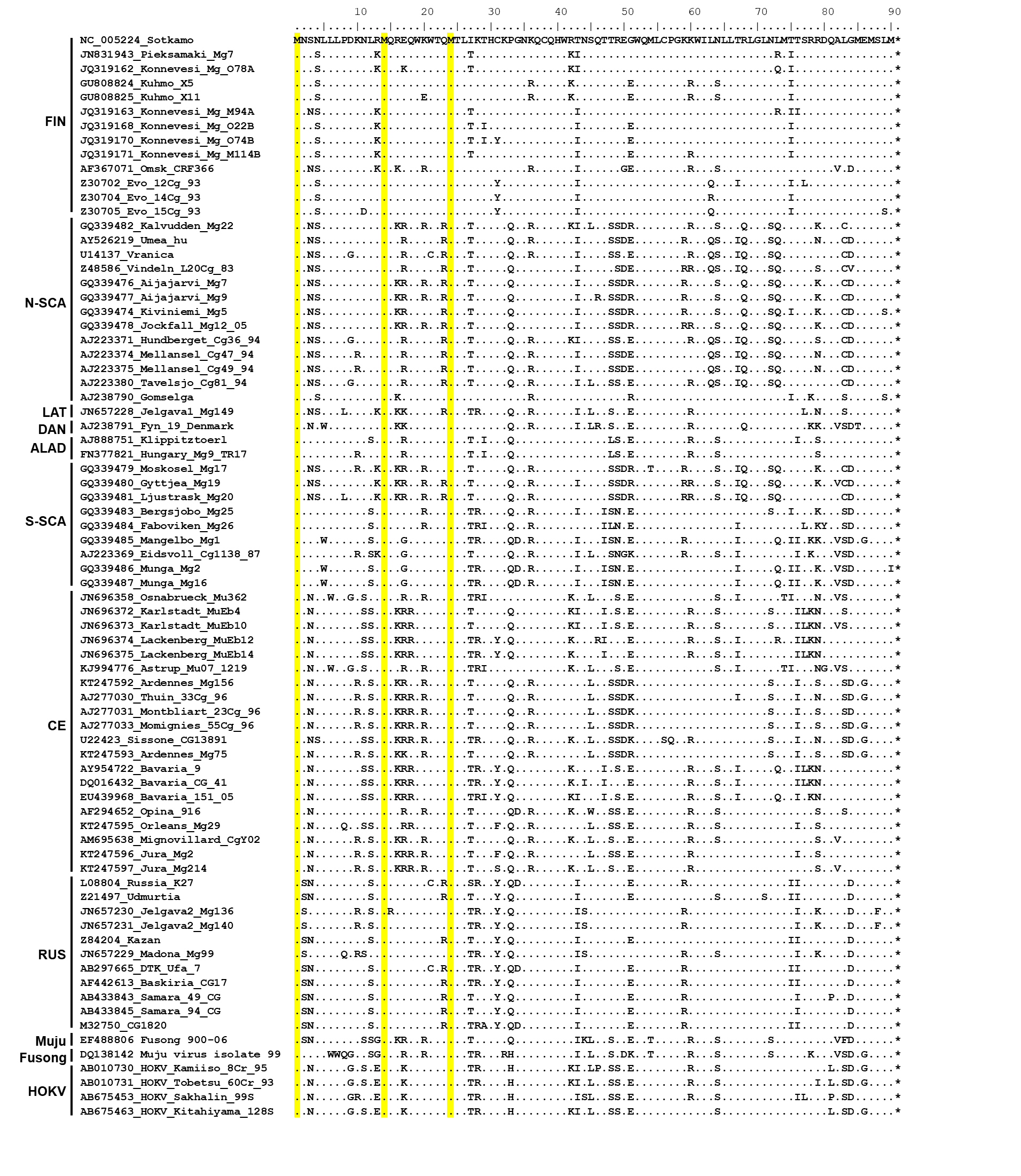
Supplementary Figure 3: Multiple alignment of the NSs protein amino acid sequences of PUUV strains of the various clades compared to the Sotkamo prototype strain.** The designation of the PUUV clades follows a previous classification (Castel et al., 2015): Alpe-Adrian (ALAD), Central European (CE), Danish (DAN), Finnish (FIN), Latvian (LAT), Northern-Scandinavian (N-SCA), Russian (RUS), Southern-Scandinavian (S-SCA) clades as well as the PUUV strains Hokkaido (HOKV), Muju, and Fusong.

**Supplementary Table 1: Summary of NSs protein expression patterns and their inhibitory activity on human interferon type I (IFN-I) promoter.**

|  | **Protein variant** | **Predicted molecular**  **mass (kDa)** | | **Observed IFN-I promoter inhibition** | |
| --- | --- | --- | --- | --- | --- |
| I | PUUV Sotkamo NSs wt | | 11.8 and 10.3 | | ++ |
|  | TULV NSs | | 12, 10.5 and 9.8 | | ++ |
|  | PHV NSs | | 11.6 and 8.9 | | ++ |
|  | KHAV NSs | | 12.4, 10.5 and 9.1 | | + |
|  | SNV NSs | | 8.7 and 7.3 | | (+) |
|  | ANDV NSs | | 8.9 and 7.5 | | (+) |
| II | PUUV KS10/972 BW | | 11.8 and 10.3 | | ++ |
|  | PUUV KS10/994 BW | | 11.8 and 10.3 | | ++ |
|  | PUUV KS10/1068 BW | | 11.8 and 10.3 | | ++ |
|  | PUUV KS12/2383 BW | | 11.8 and 10.3 | | ++ |
|  | PUUV KS15/307 BW | | 11.8 and 10.3 | | ++ |
|  | PUUV KS10/2001 NW | | 12.5 and 10.8 | | ++ |
|  | PUUV KS10/2030 NW | | 12.5 and 10.8 | | ++ |
|  | PUUV KS12/1778 NW | | 12.5 and 10.8 | | ++ |
|  | PUUV KS12/2519 NW | | 12.5 and 10.8 | | ++ |
|  | PUUV KS12/2574 BW | | 11.8 and 10.3 | | ++ |
|  | PUUV KS12/2587 BW | | 11.8 and 10.3 | | ++ |
|  | PUUV KS12/2592 BW | | 11.8 and 10.3 | | ++ |
|  | PUUV KS13/692 BW | | 11.8 and 10.3 | | ++ |
|  | PUUV Mu07/458 BW | | 11.8 and 10.3 | | ++ |
|  | PUUV Mu07/503 BW | | 11.8 and 10.3 | | ++ |
| III | PUUV Sotkamo NSs2Stop | | - | | - |
|  | PUUV Sotkamo NSs14Stop | | 8.9 | | - |
|  | PUUV Sotkamo NSs M1A | | 10.3 | | + |
|  | PUUV Sotkamo NSs M14A | | 11.8 and 8.9 | | + |
|  | PUUV Sotkamo NSs M24A | | 11.8 and 10.3 | | ++ |
|  | PUUV Sotkamo NSs M1A M14A | | 8.9 | | - |
|  | PUUV Sotkamo NSs M14A M24A | | 11.8 | | - |
|  | PUUV Sotkamo NSs M1A M14A M24A | | - | | - |
| IV | PUUV NSs KS19/16 (OS) | | 11.8 and 10.3 | | ++ |
|  | PUUV NSs KS17/1084 (OS) | | 11.8 and 10.3 | | ++ |
|  | PUUV KS19/16 NNN2-4(A)3 | | 11.8 and 10.3 | | + |
|  | PUUV KS19/16 S11A | | 11.8 and 10.3 | | ++ |
|  | PUUV KS19/16 RRQW16-19(A)4 | | 11.8 and 10.3 | | ++ |
|  | PUUV KS19/16 WTQ21-23(A)3 | | 11.8 and 10.3 | | + |
|  | PUUV KS19/16 TLTR25-28(A)4 | | 11.8 and 10.3 | | ++ |
|  | PUUV KS19/16 C31A | | 11.8 and 10.3 | | ++ |
|  | PUUV KS19/16 C38A | | 11.8 and 10.3 | | ++ |
|  | PUUV KS19/16 S49A | | 11.8 and 10.3 | | ++ |
|  | PUUV KS19/16 C56A | | 11.8 and 10.3 | | ++ |
|  |  | |  | |  |

The predicted molecular mass was calculated from the amino acid sequence using the ‘Compute pI/Mw’ tool of the Swiss Institute of Bioinformatics Resource Portal ExPASy (https://web.expasy.org/compute_pi/); BW = Baden-Wuerttemberg, IFN = interferon, kDa = kilo dalton, NW, North Rhine-Westphalia, OS = Osnabrück.

**Supplementary Table 2: Bank vole-derived PUUV NSs sequences used for functional studies on IFN-I promoter inhibition.**

| **NSs Sequence** | **Origin** | **Year** | **Accession number** | **Reference** |
| --- | --- | --- | --- | --- |
| KS19/16 | Osnabrück, LS, North-west Germany | 2018 | MN906316 | this study |
| KS17/1084 | Osnabrück, LS, North-west Germany | 2017 | MN906315 | this study |
| KS10/972 | Weissach, BW, South-west Germany | 2010 | MT453485 | Binder et al., 2020 |
| KS10/994 | Weissach, BW, South-west Germany | 2010 | MT453491 | Binder et al., 2020 |
| KS10/1068 | Weissach, BW, South-west Germany | 2010 | MT453516 | Binder et al., 2020 |
| KS12/2383 | Weissach, BW, South-west Germany | 2012 | MT453655 | Binder et al., 2020 |
| KS15/307 | Weissach, BW, South-west Germany | 2014 | MT453675 | Binder et al., 2020 |
| KS10/2001 | Billerbeck, NW, North-west Germany | 2010 | MT453553 | Binder et al., 2020 |
| KS10/2030 | Billerbeck, NW, North-west Germany | 2010 | MT453559 | Binder et al., 2020 |
| KS12/1778 | Billerbeck, NW, North-west Germany | 2012 | MT453649 | Binder et al., 2020 |
| KS12/2519 | Billerbeck, NW, North-west Germany | 2012 | MT453666 | Binder et al., 2020 |
| KS12/2574 | Steinheim, BW, South-west Germany | 2012 | MT453680 | Binder et al., 2020 |
| KS12/2587 | Stuttgart, BW, South-west Germany | 2012 | MT453682 | Binder et al., 2020 |
| KS12/2592 | Moessingen-Belsen, BW, South-west Germany | 2012 | MT453683 | Binder et al., 2020 |
| KS13/692 | Crailsheim, BW, South-west Germany | 2012 | MT453692 | Binder et al., 2020 |
| Mu07/458 | Zussdorf-Wilhelmsdorf, BW, South-west Germany | 2007 | MT453698 | Binder et al., 2020 |
| Mu07/503 | Michelbach, BW, South-west Germany | 2007 | MT453705 | Binder et al., 2020 |

LS, Lower Saxony, BW, Baden-Wuerttemberg; NW, North Rhine-Westphalia

**References**

Binder F, Ryll R, Drewes S, Jagdmann S, Reil D, Hiltbrunner M, Rosenfeld UM, Imholt C, Jacob J, Heckel G, Ulrich RG (2020) Spatial and Temporal Evolutionary Patterns in Puumala Orthohantavirus (PUUV) S Segment. Pathogens 8; 9(7): 548. doi: 10.3390/pathogens9070548. PMID: 32650456; PMCID: PMC7400055.

Castel G, Couteaudier M, Sauvage F, Pons JB, Murri S, Plyusnina A, et al. (2015) Complete Genome and Phylogeny of Puumala Hantavirus Isolates Circulating in France. Viruses 7(10): 5476-5488.
